# Supplementary material for: Patterns of Intron Gain and Loss in Fungi
Source: PLoS Biol. 2004 Nov 30;2(12):e422. doi: 10.1371/journal.pbio.0020422 (PMC532390; doi:10.1371/journal.pbio.0020422)
Supplement: Table S1 — Also available at http://genes.mit.edu/NielsenEtAl/. (4.3 MB ZIP). [file pbio.0020422.st001.zip › NielsenEtAl/html/1212.html]

AN2048.1.NCU04119.1.MG06883.1.FG01890.1


```
 CLUSTAL W (1.82) Multiple Sequence Alignments - Introns Inserted


Sequence 1: MG06883.1	357 aa
Sequence 2: FG01890.1	344 aa
Sequence 3: NCU04119.1	404 aa
Sequence 4: AN2048.1	317 aa
Alignment Length: 407 aa
Number Identitical Residues: 154 aa
Alignment Score (without introns) 7476


MG06883.1 	MWRDRTNL~YISYRQSYAHHPAKRTRYGDSSATSSSLAGADRFGGSGNSLLFSADEDRRG
NCU04119.1	MWRDRTNL2YISYRQSYAHHPTQRNRYAP-QNPGDRFGGTSGGAGSSTGVLFSADDDRRG
FG01890.1 	MWRDRTNL2YISYRQSYAHHPTQRRPYGPGASASGALA--DTYTSS------YPNEDRLG
AN2048.1  	MWRDRTNL2YISYRQSFTHHPAKKPRYLGTPNGFSDVA--------------SQSEESRR
          	******** *******::***:::  *       . ..                .::   

MG06883.1 	LLSSGAS--DDGDAVIEMDLLPPRWAEVNDDVTELLAEIAQKSQKLDRLHQKHVLPGFND
NCU04119.1	LLSAGAYDVDDGDAVIEMDLLPPRWADSSDEVAELLADIARKSQKLERLHQKHVLPGFDD
FG01890.1 	LLSAGAFE-DDGDAVIEMDVLPPRWAAFSDDITDLLADIATKGQRLEKLHQKHVLPGFND
AN2048.1  	LISESTGIDDDGDAIIEMDVLPPRWVDVQEDVTDLLADIAQKSAQLDKLHHKHLLPGFGD
          	*:* .:   *****:****:*****.  .:::::***:** *. :*::**:**:****.*

MG06883.1 	EEAKRAEESEIEDLTQQITKGFHQCHKCIQKVEHMVKDSKRTGGISRAEEVMAKNIQVSL
NCU04119.1	DEAKRDQEREIEQLTQSITKGFHDCHRVIQRIEQMVREGKHNGQMSRADEVMAKNIQINL
FG01890.1 	EEAKKAEEAQIENLTQDITKGFHACHRCIQKIEQMVRDSQHAGTITRAEETMAKNIQTSL
AN2048.1  	EEVRKQDERVIERYTQEITRGFHECQKLVKRIEVMVHEAKQQGGVSSGDETMAKNIQISL
          	:*.:: :*  **  **.**:*** *:: ::::* **::.:: * :: .:*.****** .*

MG06883.1 	ATRVQEASAGFRKKQSAYLKK1LKGMGGGGGLSPGGDRSSTPN---------PANNFTEP
NCU04119.1	ATRVQEASANFRKKQSAYLKK1LRGMSG--LASPTLDRVSATSSFLGPSGLPPGNSSNSA
FG01890.1 	ASRVQDASASFRKKQSAYLKK1LRGMGGFGALSPG-ERSSTPQ---------PGS-YLDP
AN2048.1  	ASRVQEASAQFRKKQSNYLKK1LRGLED---TASQFDRSTTPM----------QNPYTDP
          	*:***:*** ****** **** *:*: .    :.  :* ::.            .   ..

MG06883.1 	SILESDADRSFSQSTLQATQHQKLLQSNDAAILQREREIDQIAQGIIDLADLFRDLQTMV
NCU04119.1	SILESDADRVFSQSTLQASTHQKLLHSNDQAISQREREIDEIAQGIIDLSDLFRDLQTMV
FG01890.1 	SLQESDADRSFSQSTLQATQQQRVLHSNDTAIAQREREIEDIAQGIIELSDLFRDLQNMV
AN2048.1  	SLMESDADKSFSQTTLMQTT-QRMTGQNDAAILQREREINDIAKGIIELSDIFRELQAMV
          	*: *****: ***:**  :  *::  .** ** ******::**:***:*:*:**:** **

MG06883.1 	IDQGTMLDRIDYNVERMATDVKEADKELVIASG~-----------YQKKTTKRKIILLLI
NCU04119.1	IDQGTMLDRIDYNVERMATDVKEAAKELTVASG2RRNMEVEESGERHKRDELQDVMCGTI
FG01890.1 	IDQGTMLDRIDYNVERMNTDVKAADKELVVASG~-----------YQRRTTKRKIILLLI
AN2048.1  	IDQGTMLDRIDYNVERMNTDVQAAQKELN----~--------------------VVCLTT
          	***************** ***: * ***                          ::    

MG06883.1 	LIIAGMIILLIIKPKKDS~GDGG------~----------------------
NCU04119.1	ELMGGRVTETTTRYSERD1VNNRGYENRV1NSFTLDQIYFLRQGSGTSKSVG
FG01890.1 	LIIFGMIILLVIKPKKHE~----------~----------------------
AN2048.1  	LLAGIQSIELEN------~----------~----------------------
          	 :
```
